# Editorial

**Published:** 1860-12

**Authors:** 


### EDITORIAL.

### A REQUEST.

Those members of the profession who receive the present number of the *Examiner*, will place us under obligations by answering, as promptly as may be, the following questions:—

1st. How often (approximately or accurately), have you used Chloroform as an anæsthetic?

2d. Have you ever had any dangerous or fatal results from its use? If so, what?

3d. Are you personally cognizant of any dangerous or fatal results from its exhibition by others ? If so, please detail case or cases.

4th. In the detail of cases, (dangerous or fatal), be pleased to specify particularly the mode of, and *position*, of the patient during its administration, the character of the operation to be performed, alarming symptoms, restorative means used, etc.

---

### FISHER vs. STONE.

This case, which was a suit for slander instituted by Dr. A. Fisher, of this city, against H. O. Stone, recently occupied the attention of the Circuit Court through a very protracted period, and finally terminated in a verdict for the defendant.

The circumstances were briefly as follows :—Dr. Fisher, who is a physician and surgeon of good reputation and unexceptionable character, was called to attend Mrs. Stone in her confinement. The labor was rather tedious, and the delivery of the after birth accompanied by some more hemorrhage than the average, though not sufficient to create alarm. The patient was comfortably put to bed, and in a short time the attendants took some refreshments and retired. The patient progressed in her recovery slowly, but was so well at the end of nine or ten days, that the Doctor ceased his visits. She continued to progress favorably for something more than a week, during which time she sat up some, and on two or three occasions she was assisted to her piano, where she played and sang freely. About this time a moderate secondary hemorrhage occurred, which caused the patient to be confined to her bed, and to have Dr. Fisher re-called. As the hemorrhage was very moderate in amount, it was attributed to weakness and over-exertion, and the patient put upon the use of tonics and astringents, both internally and locally, without resorting to a vaginal examination. After persisting in this course for several days without success, the Doctor resorted to an examination per vaginum, and very much to his surprise found the uterus inverted and occupying the vagina. Dr. Fisher continued to attend the patient on the most friendly terms for many weeks after the discovery of the inversion, but failing to persuade her to submit to an operation for its re-position, and her health remaining bad, she determined to go to a water-cure establishment in New-York State. After she left Chicago, she soon came in contact with medical men who claim that inversion of the uterus *always* takes place during the delivery of the placenta or soon after delivery. And of course the patient and her husband were soon taught to believe that her inversion had occurred immediately after delivery, and had been either purposely concealed, or culpably overlooked by her medical attendant, Dr. Fisher.

Consequently, on his return home, Mr. Stone commenced a relentless warfare upon the professional character of Dr. Fisher. On account of this, the latter commenced the suit for slander. The defendant did not deny the slanders, but pleaded *justification*, alledging that the inversion had occurred either through the carelessness or unskilfulness of the plaintiff in delivering the placenta. A large amount of medical testimony was adduced by both parties, by which much difference of opinion was shown to exist among the most enlightened members of the profession, in relation to the mechanism of inversion of the uterus; its efficient causes, and the periods of time at which it may take place. From the opinions given in the course of this trial, and those found expressed in the pages of our medical literature, we are satisfied that there are very prevalent and important errors existing in relation to this subject. Errors, indeed, that are dangerous to the reputation of every practitioner of the obstetrical art; and which, as they now stand, afford an almost insuperable obstacle in the way of obtaining justice in any court where this subject is involved. Hence, if time will permit, we shall carefully review the subject of inversio-uteri in the next number of the *Examiner*.

---

*Resignation.*—We are informed that Prof. A. S. Hudson, has resigned the chair of Physiology and Pathology in the Rush Medical College of this city. The duties of the chair are being discharged by other members of the Faculty for the present session. The school seems to be enjoying a fair degree of prosperity, so far as regards the number of students, there being about 120 Matriculants.

---

*The Examiner.*—The present number completes the first volume of the Chicago Medical Examiner. It will continue to be issued, as heretofore, during the first week of every month. We propose to make no changes in the general plan and objects of the Journal, but to make every possible exertion to fill its pages with such matter as will be of the greatest utility to the profession of the North-West. As the January number will commence a new volume, it is a favorable time for new subscribers to send in their names. And we trust that all our old subscribers, as well as the new ones, will remember that our terms are $2 per annum, payable in *advance.*

---

*Medical Department of Lind University.*—We saw in a late number of the *American Medical Times*, a letter dated Chicago, and signed " Pillula." The writer, after giving an eulogistic account of the Chicago Charitable Eye and Ear Infirmary, and the opening of the lecture term in the Rush Medical College, states that the present session of the Lind University opened with a *rather smaller* class than last year, with the evident design to convey the impression that this Department of the University was proving a failure.

We would respectfully inform the *Times*, that " Pillula" is mistaken. The present session opened with a class twenty-five per cent. larger than last year, and the number now in regular attendance is between forty and fifty. The Junior, Senior, and clinical departments are all well attended, and in every respect the institution is enjoying a degree of prosperity fully equal to the expectations of its most sanguine friends.

